# Detection, Characterization, and Typing of Shiga Toxin-Producing *Escherichia coli*

**DOI:** 10.3389/fmicb.2016.00478

**Published:** 2016-04-12

**Authors:** Brendon D. Parsons, Nathan Zelyas, Byron M. Berenger, Linda Chui

**Affiliations:** ^1^Laboratory Medicine and Pathology, University of AlbertaEdmonton, AB, Canada; ^2^Medical Microbiology and Immunology, University of AlbertaEdmonton, AB, Canada

**Keywords:** STEC, detection, characterization, typing, O157, non-O157

## Abstract

Shiga toxin-producing *Escherichia coli* (STEC) are responsible for gastrointestinal diseases reported in numerous outbreaks around the world. Given the public health importance of STEC, effective detection, characterization and typing is critical to any medical laboratory system. While non-O157 serotypes account for the majority of STEC infections, frontline microbiology laboratories may only screen for STEC using O157-specific agar-based methods. As a result, non-O157 STEC infections are significantly under-reported. This review discusses recent advances on the detection, characterization and typing of STEC with emphasis on work performed at the Alberta Provincial Laboratory for Public Health (ProvLab). Candidates for the detection of all STEC serotypes include chromogenic agars, enzyme immunoassays (EIA) and quantitative real time polymerase chain reaction (qPCR). Culture methods allow further characterization of isolates, whereas qPCR provides the greatest sensitivity and specificity, followed by EIA. The virulence gene profiles using PCR arrays and stx gene subtypes can subsequently be determined. Different non-O157 serotypes exhibit markedly different virulence gene profiles and a greater prevalence of stx1 than stx2 subtypes compared to O157:H7 isolates. Finally, recent innovations in whole genome sequencing (WGS) have allowed it to emerge as a candidate for the characterization and typing of STEC in diagnostic surveillance isolates. Methods of whole genome analysis such as single nucleotide polymorphisms and k-mer analysis are concordant with epidemiological data and standard typing methods, such as pulsed-field gel electrophoresis and multiple-locus variable number tandem repeat analysis while offering additional strain differentiation. Together these findings highlight improved strategies for STEC detection using currently available systems and the development of novel approaches for future surveillance.

## Introduction

Shiga toxin-producing *Escherichia coli* (STEC) encompass a heterogeneous group of enteric pathogens responsible for numerous sporadic infections and large outbreaks worldwide. Accurate and rapid diagnosis of STEC infections is important for the appropriate management of infected patients and for implementation of proper public health interventions. Specifically, patients infected with STEC should not be treated with antibiotics because of the risk of developing hemolytic uremic syndrome (HUS) (Wong et al., 2000, 2012; Smith et al., 2012). Also, once STEC is identified in a patient, the contacts and potential sources of infection must be identified to prevent further spread of the disease. Although, laboratories have become proficient at detecting O157:H7 infections, they often do not screen stools for other STEC serotypes. This creates a gap in diagnostics; since 50% or more of STEC infections may be caused by non-O157 STEC, our surveillance and understanding of the epidemiology of STEC disease is incomplete (Fey et al., 2000; Jelacic et al., 2003; Thompson et al., 2005; Chui et al., 2011; Couturier et al., 2011; Scallan et al., 2011; Gould et al., 2013; STEC, National Surveillance Summary, 2014).

Growing recognition of the shortfall in STEC detection has prompted a shift toward more comprehensive STEC identification methods. Bacteriological culture remains the gold standard test, given the importance of identifying viable bacterial isolates for typing. For this reason, there has been increased development and use of agars which also select for non-O157 STEC (Kase et al., 2015). However, as culture-based methods are laborious and exhibit clear limits in sensitivity for STEC detection, it is recommended that laboratories supplement culture-based approaches with other assay types (Gould et al., 2009).

Alongside culture-based STEC testing many laboratories assay for the presence of Shiga toxins (Stx) or the *stx* genes. Shiga toxin was originally referred to as verotoxin for its cytotoxic effect on Vero cells (Konowalchuk et al., 1977). Once Stx was linked to hemorrhagic colitis and HUS, researchers developed cytotoxicity assays to detect Stx from both fecal specimens as well as from enriched stool cultures containing polymyxin B (Karmali et al., 1985). While such laborious cytotoxicity assays remain a method of diagnosis for some laboratories, detection of Stx or the presence of the *stx* genes is now primarily done in clinical laboratories by enzyme immune assay (EIA) of some form, or by polymerase chain reaction (PCR)-based approaches, respectively. These methods can also determine if Stx1 or 2 are present, which adds prognostic value, since there is a well-documented correlation of Stx2 with the clinical severity of STEC infection and the risk of HUS (Schimmer et al., 2008; Soon et al., 2013; Chui et al., 2015a).

While advancements in the sensitivity and speed of STEC detection have direct implication on the diagnosis and treatment of diarrheal illnesses, characterization of STEC isolates beyond serotype or individual virulence factors is required for prevention, control and prediction of STEC infections on a public health scale. The requirement for high resolution typing of STEC is also increasingly necessary given the observed emergence of diverse types of virulent strains (Soon et al., 2013). Current STEC fingerprinting techniques such as pulsed-field gel electrophoresis (PFGE) or multi-locus variable number tandem repeat analysis (MLVA) using the PulseNet International protocol allow comparison of strains from different countries and aid in the epidemiological tracking of STEC infections around the world, especially during outbreak settings (Sabat et al., 2013). While methods such as PFGE and MLVA play a crucial role in current outbreak investigations, the increasingly tractable use of whole genome sequencing (WGS) has garnered significant interest as a powerful method for typing bacterial pathogens (Chattaway et al., 2016). WGS technologies promise typing resolution at orders of magnitude greater than existing methods. Yet, as technical capabilities improve for STEC typing, new challenges arise surrounding implementation, standardization and management of typing data (Köser et al., 2012; Franz et al., 2014).

Here we present an overview of recent advances and the experiences at the Provincial Laboratory of Public Health (ProvLab) in Alberta Canada with various bacteriological, molecular and genomic strategies for detection and typing of STEC. We assess the benefits and shortcomings of various methods used in the detection and differentiation of STEC. Through evaluation of available systems and opportunities for novel approaches, this review aims to identify improved strategies for STEC identification and surveillance.

## Differential and selective media

When *E. coli* O157:H7 was first identified as an etiologic agent of hemorrhagic colitis, it was discovered to be unlike most other strains of *E. coli*, because it could not ferment sorbitol (Wells et al., 1983; Pai et al., 1984). This biochemical peculiarity led to the use of sorbitol-MacConkey (SMAC) agar to identify non-sorbitol fermenting *E. coli* in stool of patients with bloody diarrhea (Remis, 1984). This agar differed from typical MacConkey agar by substituting lactose with sorbitol; non-sorbitol fermenting organisms produced white colonies on the medium (Remis, 1984). Early investigations found that SMAC agar displayed acceptable sensitivity, specificity, and negative predictive value (NPV) for *E. coli* O157:H7 detection, but a positive predictive value (PPV) of only 28% (Table 1; March and Ratnam, 1986). Besides its low PPV, other limitations of SMAC include its inability to detect non-O157 STEC as well as sorbitol-fermenting O157 STEC isolates, which can carry the toxigenic *stx* genes and cause outbreaks (Gunzer et al., 1992; Ammon et al., 1999). To improve the detection of STEC, a new chromogenic medium, CHROMagar™ O157, was developed by CHROMagar Microbiology (CHROMagar™ O157 CHROMagar Microbiology, Paris, France, 2013). Through the incorporation of proprietary chromogenic substrates in CHROMagar™ O157 agar, O157 STEC appear mauve while other *E. coli* are blue. Unfortunately, as with SMAC agar, CHROMagar™ O157 is not able to detect most non-O157 STEC (Bettelheim, 1998). The only study directly comparing SMAC agar to CHROMagar™ O157 showed that CHROMagar™ O157 had a lower false-positive rate for colony picks (20%) compared to that of SMAC agar (65%) as well as estimated annual cost-savings of approximately $76,000 in a large Canadian clinical laboratory (Church et al., 2007). In the same study, CHROMagar™ O157 had a higher sensitivity (96.3% vs. 85.2%) for detecting O157 STEC (Table 1; Church et al., 2007). Other chromogenic media, such as Colorex™ O157 (Alere, Inc., Ottawa, ON, Canada), are also available to detect O157 STEC, but have not been clinically evaluated.

**Table 1:** **Summary of clinically evaluated STEC detection methods**.

**Assay**	**Description**	**Sensitivity/Specificity**	**PPV/NPV**	**References**
**SOLID MEDIA**				
CHROMagar™ O104 STEC	Detects O104:H4 STEC through chromogenic substrate	71.4%/99.1%	62.5%/99.4%	Gouali et al., 2013
CHROMagar™ O157	Detects O157 STEC through a chromogenic substrate	96.3%/100%	100%/100%	Church et al., 2007
CHROMagar™ STEC	Detects O157 and non-O157 STEC through a chromogenic substrate	84.6–85.7%/87–95.8%	13.9–60%/98.9–99.6%	Wylie et al., 2013; Zelyas et al., 2016
Rainbow® Agar O157	Detects O157 and some non-O157 STEC through β-glucuronidase and β-galactosidase activities	Not determined	Not determined	Zelyas et al., 2016
Sorbitol-MacConkey	Detects O157 STEC by lack of sorbitol fermentation	85.2–100%/85–100%	28–100%/99.9–100%	March and Ratnam, 1986; Church et al., 2007
**TOXIN DETECTION**				
BioStar SHIGATOX	Optical immunoassay detects and differentiates Stx1/2	96.8%/99.4%	88.2%/99.9%	Church et al., 2007
Duopath Verotoxin-test™	Immunochromatographic assay detects and differentiates Stx1/2	88.9%/71.4%	42.1%/96.5%	Grif et al., 2007
Immunocard STAT!®	Immunochromatographic assay detects and differentiates Stx1/2	35.5%/99.4%	54.5%/98.6%	Chui et al., 2013
Premier® EHEC	Microwell EIA detects Stx1/2 without toxin differentiation	90.5–96.8%/98.5–100%	76.2–100%/98–99.9%	Grif et al., 2007; Teel et al., 2007; Chui et al., 2011; Hermos et al., 2011
ProSpect™ Shiga Toxin *E. coli*	Microwell EIA detects Stx1/2 without toxin differentiation	76.8%/100%	100%/96.1%	Gerritzen et al., 2011
Shiga Toxin Chek™	Microwell EIA detects Stx1/2 without toxin differentiation	80%/98.2%	53.3%/99.5%	Chui et al., 2015b
Shiga Toxin Quik Chek™	Immunochromatographic assay detects and differentiates Stx1/2	85%/100%	100%/99.6%	Chui et al., 2015b
**MOLECULAR DETECTION**				
EntericBio real-time Gastro Panel I®	Multiplex PCR detects four pathogens including STEC	100%/99.8%	82.9%/100%	Koziel et al., 2013
FilmArray® GI panel	Array-based multiplex PCR detects 22 pathogens including STEC	100%/99.1–99.6%	86.8–95.1%/100%	Khare et al., 2014; Buss et al., 2015
Seeplex® Diarrhea ACE Detection	Multiplex PCR with dual priming oligonucleotide technology detecting 14 pathogens including STEC	100%/99.6–100%	66.7–100%/100%	Coupland et al., 2013; Onori et al., 2014
TaqMan® in-house STEC detection assay	Real-time PCR assay developed by Alberta ProvLab detecting *stx*_1_ and *stx*_2_ separately	100%/100%	100%/100%	Chui et al., 2010
xTag® Gastrointestinal Pathogen Panel	Multiplex PCR with bead hybridization detecting 15 pathogens including STEC	93.5–100%/98.8–100%	75–100%/99.1–100%	Claas et al., 2013; Navidad et al., 2013; Deng et al., 2015

PPV, positive predictive value; NPV, negative predictive value; for CHROMagar™ O104 STEC, diagnostic values are for O104:H4 STEC only; for CHROMagar™ O157 and sorbitol-MacConkey, diagnostic values are for O157:H7 STEC only; for all other assays, diagnostic values are for all STEC serotypes.

Many clinical microbiology laboratories continue to use either SMAC agar or CHROMagar™ O157 exclusively to detect STEC even though they are not appropriate for the detection of non-O157 STEC. Guidelines released by the Centers for Disease Control and Prevention (CDC) specify that laboratories are to simultaneously culture stool specimens for O157 STEC and test them with an assay that detects non-O157 STEC (Gould et al., 2009). Because of the shortcomings of SMAC and CHROMagar™ O157, there has been interest in creating a medium capable of detecting non-O157 serotypes of STEC.

During the 2011 *E. coli* O104:H4 outbreak in Europe, a medium specifically designed to detect the outbreak strain, CHROMagar™ STEC O104, was developed (Gouali et al., 2013). While this agar is able to detect the O104:H4 strain expressing an extended-spectrum β-lactamase (ESBL) initially causing the outbreak, it is unable to detect other non-O157 serotypes and O104:H4 isolates that have lost the plasmid encoding the ESBL (Mariani-Kurkdjian et al., 2011; Grad et al., 2012). Its utility is also hampered by low sensitivity and PPV (Table 1).

Other chromogenic agars capable of detecting wider ranges of STEC serotypes have been described, including Rainbow® Agar O157 and CHROMagar™ STEC (Biolog, Hayward, CA, USA, 2008; CHROMagar™ STEC, CHROMagar Microbiology, Paris, France, 2014). CHROMagar™ STEC is meant to detect all STEC serotypes. Like other media developed by CHROMagar Microbiology, pathogen detection on CHROMagar™ STEC is based on the organism's utilization of proprietary chromogenic substrates. CHROMagar™ STEC is able to detect most of the STEC serotypes for which it has been assessed (Hirvonen et al., 2012; Wylie et al., 2013; Zelyas et al., 2016). Direct inoculation of stool onto the agar yields acceptable sensitivity, specificity, and NPV, but the PPV is quite low (Wylie et al., 2013; Zelyas et al., 2016). Two studies using different broth enrichment protocols prior to inoculation showed sensitivities varying from 50% (McCallum et al., 2013) to 91.4% (Gouali et al., 2013).

Rainbow® Agar O157 is purported to detect O157:H7, O26:H11, O48:H21, O111:H-, and O111:H8 serotypes based on their reduced or absent β-glucuronidase activity compared to non-toxigenic strains (Biolog, Hayward, CA, USA, 2008). Although, Rainbow®Agar O157 has been evaluated in a number of studies for the detection of STEC in food and water (Radu et al., 2000; Tutenel, 2003; Tillman et al., 2012; Yoshitomi et al., 2012; Ngwa et al., 2013), its ability to identify STEC from human stool was first investigated at the Alberta ProvLab. A study by Zelyas et al. (2016) performed at the Alberta ProvLab compared four chromogenic agar media in their ability to detect non-O157 STEC. Isolates from a panel of 161 non-O157 STEC were inoculated directly onto CHROMagar™ STEC, Rainbow® Agar O157, CHROMagar™ O157, and Colorex® O157 to observe if the isolates would produce STEC-like colonies. Unsurprisingly, CHROMagar™ O157 and Colorex® O157 were unable to identify the majority of non-O157 isolates as STEC, while CHROMagar™ STEC and Rainbow® Agar O157 had detection rates of 90% and 70%, respectively. Using stool cultures spiked with non-O157 STEC isolates, it was found that CHROMagar™ STEC once again exhibited a superior detection rate of 72% (compared to 26% using Rainbow® Agar O157) in bloody stool. Similar to previous studies, CHROMagar™ STEC demonstrated a sensitivity, specificity, PPV, and NPV of 84.6, 87, 13.9, and 99.6%, respectively, when 536 clinical specimens were inoculated directly onto the medium (Table 1). Although, studies demonstrate that CHROMagar™ STEC shows promise in its ability to rule out STEC in its absence, the high number of false-positive results seen on the medium would necessitate considerable additional laboratory testing to confirm or deny STEC status of mauve colonies. The use of CHROMagar™ STEC should perhaps be limited to the procurement of STEC isolates when a stool tests positive for STEC by a non-culture method, such as toxin or toxin gene detection. As discussed below, such non-culture methods often display sensitivities above the ~85% seen with CHROMagar™ STEC.

## Enzyme immunoassays

As no culture medium is yet available for the practical detection of all STEC serotypes, identifying the Shiga toxin (Stx) in stools is an alternative method of diagnosing STEC-related disease. The first EIAs developed for Stx identified STEC colonies based on the binding of monoclonal antibodies to Stx1 and Stx2 immobilized on membranes (Perera et al., 1988; Milley and Sekla, 1993). Since the creation of these early EIAs that required the growth of isolated colonies on a solid medium, a number of other assays have been developed for the detection of Shiga toxin directly from stool or from enriched stool cultures.

One of the most evaluated and used EIAs is the Premier® EHEC microwell immunoassay (Meridian Bioscience Inc., Cincinnati, OH, USA). Multiple studies using overnight broth enrichment stool cultures found that Premier® EHEC demonstrates high sensitivity and specificity (Table 1). Premier® EHEC has also been used to detect Stx directly from clinical specimens without the use of an overnight enrichment step; one group found this approach had a sensitivity of 83.9% and specificity of 99.8% (Teel et al., 2007). Another microwell immunoassay that has undergone clinical evaluation, the ProSpect™ Shiga Toxin *E. coli* assay (Remel, Lenexa, KS, USA), demonstrated inferior sensitivity compared to Premier® EHEC (Table 1).

Besides microwell EIAs, other types of immunoassays have been developed to detect STEC. One such assay is the BioStar® SHIGATOX optical immunoassay (Inverness Medical Professional Diagnostics, Inc., San Diego, CA, USA) which detects Stx by its interaction with anti-Stx antibodies on the surface of a silicon wafer; this interaction causes an increase in the optical thickness of the thin film and results in a visible color change on the wafer. Similarly, the Duopath Verotoxin-test™ (Merck, Darmstadt, Germany) is an immunochromatographic assay that employs anti-Stx antibodies immobilized to a membrane to bind and detect Stx. In previous studies, the BioStar® SHIGATOX assay exhibited a superior performance to the Duopath Verotoxin-test™ (Table 1). However, the Duopath Verotoxin-test™ is advantageous because it differentiates between Stx1- and Stx2-producing STEC.

Studies performed at the Alberta ProvLab have evaluated two microwell immunoassays: the aforementioned Premier® EHEC and the Shiga Toxin Chek™ assay (TechLab, Inc., Blacksburg, VA, USA; Chui et al., 2011, 2015b). Premier® EHEC demonstrated a sensitivity of 90.5%, similar to that seen in previous studies (Grif et al., 2007; Teel et al., 2007; Hermos et al., 2011), and the Shiga Toxin Chek™ assay had a lower sensitivity of 80% which decreased to 70% when unenriched specimens were used (Table 1; Chui et al., 2011, 2015b).

Additionally, two immunochromatographic assays have been assessed at the Alberta ProvLab: ImmunoCard STAT!® (Meridian Bioscience, Inc., Cincinnati, OH, USA) and Shiga Toxin Quik Chek™ (TechLab, Inc., Blacksburg, VA, USA; Chui et al., 2015b, 2013). Despite having a specificity >99%, ImmunoCard STAT!® had a low sensitivity of 35.5% even when using enrichment broths (Table 1). Shiga Toxin Quik Chek™ demonstrated sensitivities of 85 and 70% with and without enrichment, respectively (Chui et al., 2013, 2015b).

Some caution must be exercised when using EIAs alone to detect STEC. There have been two norovirus outbreaks in the United States in which EIAs yielded false-positive STEC results, highlighting the pitfall of depending on a single method to diagnose STEC-related disease (Centers for Disease Control and Prevention (CDC), 2001, 2006).

## Molecular methods

While the detection of Stx is a direct way to determine if clinical specimens harbor STEC, there has been much interest in nucleic acid-based methods to detect the presence of the *stx* genes in stools. The earliest application of nucleic acid detection for STEC involved the use of cloned portions of *stx*_1_ and *stx*_2_ as ^35^S-labeled DNA probes in colony hybridization assays (Willshaw et al., 1987; Scotland et al., 1988). Soon after DNA hybridization assays were developed, a conventional PCR targeting *stx*_1_ and *stx*_2_ in a single reaction was devised (Pollard et al., 1990); a similar assay was later described which could detect STEC from DNA isolated from stool (Brian et al., 1992).

A multitude of PCR assays have been developed since and a number of them use real-time platforms. Some of the advantages of using real-time PCR assays include excellent sensitivity and specificity and the ability to devise multiplex assays to detect and differentiate between *stx*_1_ and *stx*_2_, other virulence genes such as the intimin gene, *eae*, and hemolysin gene, *ehx4*, and even other gastrointestinal pathogens. The first reported *stx*-targeting real-time PCR assay used directly on naturally infected clinical stool had a sensitivity of 100% and a specificity of 92% (Bélanger et al., 2002). Numerous real-time PCR assays have been designed and generally demonstrate similarly high detection rates with few false positive results (Grys et al., 2009; Gerritzen et al., 2011; Zhang et al., 2012). Commercial real-time PCR assays such as the GeneDisc® (GeneDisc® Technolgies Pall Corporation, NY, USA) and BAX® System (DuPont Nutrition and Health, Wilmington, DE, USA) include a panel for rapidly screening for STEC, targeting *stx*_1_, *stx*_2_, and *eae* or other genes, followed by panels that target serotype-specific genes of O157 STEC and top six non-O157 STEC. These real-time PCR STEC panels exhibit high sensitivity and can be applied in two step screening algorithms that first capture STEC followed by detection the most frequently reported STEC serotypes (Fratamico et al., 2012; Wasilenko et al., 2014) Most real-time PCR assays use any one of a number of available detection systems, including SYBR green, TaqMan®, molecular beacon probes, fluorescence resonance energy transfer (FRET) probes, LUX™ (light upon extension) assays with singly-labeled primers without probes, as well as other methods. Further contributing to the heterogeneity of available methods, different real-time PCR assays often target different regions within *stx*_1_ and *stx*_2_, (Chui et al., 2010).

Numerous multiplex molecular assays for the detection of multiple gastrointestinal pathogens are also available. The xTag® Gastrointestinal Pathogen Panel (GPP) (Luminex Corporation, Austin, TX, USA), is FDA- and Health Canada-approved for the detection of multiple agents of gastroenteritis. The GPP employs a multiplex PCR with a reverse transcriptase step intended to amplify nucleic acid from nine bacterial pathogens, three parasites, and three viruses. The generated amplicons are then hybridized to oligonucleotides bound to microspheres, which are detected by the instrument. Included in the GPP are separate targets for the detection of *E. coli* O157 and non-O157 STEC. Multiple evaluations performed in different regions have demonstrated high sensitivities and specificities (Table 1). Some studies report the detection of STEC by the GPP in culture- or conventional PCR-negative specimens; the significance of these results, whether due to heightened sensitivity of the GPP or to false-positives, has not been determined (Mengelle et al., 2013; Vocale et al., 2015). The EntericBio real-time Gastro Panel I® (Serosep, Limerick, Ireland), the FilmArray® GI panel (BioFire, Inc., Salt Lake City, UT, USA), and the Seeplex® Diarrhea ACE Detection system (Seegene, Seoul, South Korea) demonstrate similar sensitivities and specificities for STEC as the GPP (Table 1).

A method of considerable interest that has yet to be clinically evaluated for the detection of STEC from human stool is loop-mediated isothermal amplification (LAMP). The assay uses a DNA polymerase with strand-displacement activity and four to six specially-designed primers to generate high numbers of stem-loop amplicons in as little as 1 h at a stable temperature of 60–65°C; real-time visualization of positive reactions occurs with the production of insoluble magnesium pyrophosphate, thus obviating the need for fluorescent reporters (Notomi et al., 2000; Mori and Notomi, 2009). Advantages of LAMP include a high sensitivity and specificity, short turn-around-time, isothermal conditions, and a simple detection method. Two studies employed LAMP to detect STEC from human stool thus far, neither determined sensitivities or specificities for the assays used (Wang et al., 2012; Teh et al., 2014). However, a LAMP assay developed by Hara-Kudo et al. (2007) to detect STEC, had 100% sensitivity for *stx*_**1**_ and *stx*_**2**_, and a specificity of 98% for *stx*_**1**_ and 100% for *stx*_**2**_ in tests of stool samples at the Alberta ProvLab. As well, the PPV for this assay was 92% for *stx*_**1**_and 100% for *stx*_**2**_ while both *stx*_**1**_ and *stx*_**2**_ had an NPV of 100%. Although, a major disadvantage of LAMP is the difficulty in developing multiplex assays, one could envision how the advantages of the LAMP assay could be exploited in point-of-care testing. However as of yet, more clinical evaluations are needed.

The Alberta ProvLab compared the diagnostic characteristics and costs associated with five PCR assays (Chui et al., 2010). This analysis showed that an in-house assay using the TaqMan® platform with a rapid turn-around-time costs the least among the real-time PCR assays, making it the most attractive test (Table 1; Chui et al., 2010). This assay has been used by ProvLab to determine the prevalence of STEC infections in various areas of Alberta during 2006–2012 as well as to act as a comparator for other methods of STEC detection (Chui et al., 2011, 2015b, 2013; Couturier et al., 2011; Zelyas et al., 2016). As well, the Alberta ProvLab is participating in the APPETITE (Alberta Provincial Pediatric EnTeric Infection TEam) study, which is comparing the GPP to routine detection methods in a large pediatric cohort from 2014 to 2019 to better define the epidemiology of gastrointestinal disease in Alberta (Freedman et al., 2015). Since routine STEC detection methods in Alberta currently involve only the identification of O157:H7 STEC through culture methods, the use of the GPP during the APPETITE study will greatly enhance STEC disease detection among children with acute gastroenteritis and serve to further evaluate the diagnostic value of the GPP.

## An algorithm to maximize STEC detection

None of the aforementioned approaches is without drawbacks. Culture techniques either lack sensitivity or a robust PPV, toxin detection assays may yield false-positives and are often expensive, and molecular methods tend to be laborious and/or expensive. At the same time, each method has at least one advantage: culture allows the isolation of strains for typing; EIAs confirm the production of disease-causing toxin and permit non-O157 STEC to be detected; and current nucleic acid tests have high sensitivity and specificity for all STEC serotypes. As suggested by the CDC in 2009, STEC detection algorithms are of most utility if a combination of culture and non-culture methods are used (Gould et al., 2009). One approach would be to pool clinical specimens and test them initially using a non-culture method. This would have the benefit of keeping costs low while screening for a low-prevalence disease. Once a positive result is obtained, the individual stools could be tested by the same non-culture method to identify the STEC-positive specimen (Chou et al., 2014). This would be followed by culture of the specimen on a chromogenic agar to obtain the isolate for typing.

While the design of detection and characterization algorithms may use various combinations of testing methods, the ultimate goal for public health investigations of STEC are aimed at specifically identifying “pathogenic” strains of STEC. However, strategies to detect pathogenic STEC are hindered by a lack of a consistent association between any single marker or combination of markers and the severity of disease. Essentially, there exist no absolute characteristic of pathogenic STEC, and therefore testing algorithms need sufficient inclusivity to capture emerging strains. As outlined by the 2013 European Food Safety Authority (EFSA) criteria for assessing STEC pathogenicity, the O104 outbreak in 2011 revealed significant shortfalls in previous testing algorithms. Specifically, algorithms that focus on identification of a narrow panel of serogroups, virulence genes or reliance on seropathotypes, which define the reported frequencies of certain serotypes with human disease, are likely insufficient to detect “non-typical” emerging STEC strains (EFSA BIOHAZ Panel, 2013). As such, the O104 outbreak strain was not included in any seropathotype category prior to 2011, and screening strategies at the time required detection of *stx* and *eae* before attempting to isolate the suspect STEC, therefore missing O104, which was *eae* negative. Modifications to STEC detection algorithms outlined by the EFSA include requirements to attempt isolation of STEC from all samples positive for *stx* genes. In addition, the EFSA panel also recommended testing for the presence of *aaiC* (a secreted protein of EAEC) and *aggR* (a plasmid-encoded regulator) genes associated with enteroaggregative adhesion, which along with *stx* and *eae* exhibit higher associated risk of severe disease. The continued improvements in STEC identification and strategic testing algorithms will aid epidemiological investigations and provide early detection of future STEC outbreaks.

## Genomics and genotyping in surveillance

Once STEC is identified and isolated in culture, the next challenge is to identify the relatedness of isolates for the purpose of public health surveillance. As discussed, STEC isolates can be classified initially based on the serotype, but additional typing is required to determine if an isolate is related to another of the same serotype. PFGE has been used extensively in public health to determine the relatedness of isolates of many bacterial species including STEC. For STEC, enhanced resolution can be achieved by combining PFGE with MLVA or using PFGE alone. Networks of public health laboratories accredited to run PFGE and/or MLVA report to their regional PulseNet organization the profiles of organisms they type (i.e., PulseNet International). This facilitates identifying national or international outbreaks that would otherwise go unnoticed. In our laboratory, this process through PulseNet Canada and PulseNet USA has found cases linked to Albertan foodborne outbreaks associated with the cross-border trade of food products between different provinces of Canada and the two countries (internal communications).

PFGE and MLVA have great utility in outbreak investigations of STEC and are advantageous because they are amenable to intra-laboratory comparison. However, for many types of bacteria, these methods and others do not have adequate resolution to identify outbreaks. WGS, on the other hand, has been demonstrated in numerous cases to provide enhanced resolution compared to pre-WGS methods (Gilchrist et al., 2015) because the entire genome (or most of the genome) can be analyzed rather than just one or a few genetic elements. In comparison to the current STEC typing and epidemiological screening methods, WGS has superior discriminatory power for comparable cost and would dramatically streamline the detection and typing workflow by replacing the multiple tests required for current investigations (Joensen et al., 2014; Dallman et al., 2015a,b).

## Whole genome sequencing

Before discussing the studies demonstrating the utility of WGS for STEC typing, one must be aware that multiple computing methods exist to assess relatedness between a set of isolates. These methods can be broadly categorized into those that analyze the difference in single nucleotide variants (SNV) between isolates (also referred to as single nucleotide polymorphisms [SNP]), nucleotide differences, gene presence or absence throughout the whole genome, gene allele differences, or overall genetic similarity (e.g., *k*-mers, average nucleotide identity, and multiple genome alignment; Konstantinidis et al., 2006; Sims et al., 2009; Nielsen et al., 2011; Maiden et al., 2013; Leekitcharoenphon et al., 2014). Once sequenced using a next-generation sequencer such the IonTorrent™ (Thermo Fisher Scientific, Waltham, USA)^1^ or HiSeq™/MiSeq™ (Illumina Inc., San Diego, USA)^2^ platforms, one or two files are generated depending on if single-end or paired sequencing is done, respectively. The file(s) contain all the “raw” sequence reads of the genomic fragments of ~150–300 bp in length, which can then be analyzed directly or assembled into contigs to form a draft genome. The assembly can be done with the help of a reference genome (e.g., *E. coli* O157:H7 *str.* Sakai) or *de novo.* Most methods used to demonstrate the utility of WGS in public health investigation of bacteria have used assembly-based analysis. In general, assembly uses one of more than 10 assemblers available, but microbiological investigations, especially for *Enterobacteriaceae*, have generally used Velvet or Burrows-Wheeler Aligner (Zerbino and Birney, 2008; Li and Durbin, 2009). Once assembled, the genome can then be compared to other genomes to identify similarity. This is the step in which there are the diverse aforementioned analysis methods with each having multiple different software algorithms and parameters in which to approach them. These approaches are bundled into analysis “pipelines” in which a raw or assembled genome sequence file can be inputted and subjected to multiple software algorithms with specified settings (Kisand and Lettieri, 2013).

Most genome analysis pipelines require high performance computing. There are, however, commercial methods emerging that have optimized genome assembly and analysis to run on high-performance desktop computers or utilize external computing infrastructure to run the computationally intensive steps. These commercial methods include: Bionumerics (Applied Maths NV, Sint-Martens-Latem, Belgium)^3^, CLC Genomic Workbench (Qiagen, Redwood City, CA, USA) and Ridom™ Seqsphere+ (Ridom GmbH, Münster, Germany)^4^. For SNV analyses, there are also online web-interfaces that allow the user to upload a set of sequence files and use a genomic center's computing infrastructure to run the software (e.g., SeqSero [http://www.denglab.info/SeqSero] and the Center for Genomic Epidemiology [http://www.genomicepidemiology.org]).

## WGS in STEC surveillance

SNV analysis has been the predominant method used to date to type isolates using WGS. Usually only the portion of the genome that is conserved amongst a species or a specific pathovar is used for determining strain relatedness (Tettelin et al., 2005; Maiden et al., 2013). This type of analysis is coined “core SNV” analysis to differentiate from SNV analysis of the entire genome. Four groups have applied SNV analysis for the typing of STEC for clinical public health purposes: ProvLab in collaboration with PulseNet Canada and the Public Health Agency of Canada National Microbiology Laboratory (PHAC-NML), the Danish Center for Genomic Epidemiology, Health Protection Scotland and Public Health England.

The Serum Staten Institute in Copenhagen, Denmark sequenced 42 isolates received in a 7-week period and determined their relationship using their web-based tools SNPtree and NDtree (Joensen et al., 2014). During this study period they had an outbreak with 13 cases of *E. coli* O157:H7, six of which were included in their study. The NDtree method, an assembly-free approach that compares the test isolates to nucleotide segments of a reference genome and generates a score representing the differences in nucleotides found between the genomes, was able to distinguish the outbreak O157 isolates from the other six O157 non-outbreak isolates and the other STEC serotypes. The SNPtree method clustered all serotypes except for O117 K1:H7 together and found 29–65 SNVs different within the outbreak O157 isolates and 521–753 SNVs different between the outbreak and non-outbreak O157 isolates. This group also demonstrated the ability of SNPtree and NDtree methods to differentiate *Salmonella* Typhimurium strains from each other (Leekitcharoenphon et al., 2014).

Public Health England has demonstrated that SNV phylogenetic methods can accurately identify outbreak isolates while adding increased sensitivity to current methods (MLVA and epidemiological investigations in this case; Dallman et al., 2015a). In one of their studies, 572 isolates received by the Gastrointestinal Bacterial Reference Unit for typing including randomly selected isolates from 2012 (*n* = 334) and 2013 (*n* = 147) were sequenced. Based on temporal and epidemiological linkages, the maximum number of SNV differences for isolates to be part of the same cluster was found to be five. An intriguing part of this study was that SNV analysis identified two outbreaks that were not detected by their routine epidemiological investigations or MLVA typing, but were later found to have previously unrecognized epidemiological linkages. This group also demonstrated that SNV analysis was concordant with epidemiological investigations in its ability to identify two different outbreaks caused by watercress contaminated with *E. coli* O157 from two different retailers (one supplied by imports from North America and Europe and the other supplied from south England; Jenkins et al., 2015). SNV analysis may also be applicable to non-O157 because in one study of a nursery school-associated *E. coli* O26:H11 outbreak, ≤3 SNVs differences were found in outbreak associated-isolates compared to ≥272 SNVs differences between outbreak and non-outbreak isolates (Dallman et al., 2015b).

In Scotland, using SNV methods, a 5-year retrospective review of 105 *E. coli* O157 isolates and 11 epidemiologically linked clusters, found that WGS was generally concordant with MLVA (Holmes et al., 2015). In this study, epidemiologically linked cases exhibited SNV differences of ≤4, while unrelated cases had SNV differences between 9 and 1632. Two sets of isolates that differed in only one MLVA locus were 32 and 126 SNVs different from the other isolate in each set, demonstrating the increased discriminatory power of WGS.

In 2014, Alberta had one of the largest outbreaks of *E. coli* O157:H7 since monitoring by PulseNet Canada began in 2000 with a final tally of 119 clinical cases, which was linked to pork consumption (ProMed-mail post 2759887, 2014). The PHAC-NML SNVPhyl pipeline was used to detect SNV in 111 of these clinical cases and 6 environmental/food isolates and was compared to the current protocol for identifying outbreaks, which involves PFGE and MLVA profiling in combination with epidemiological investigations (Sabat et al., 2013). Clinical, food and environmental isolates from the pork-associated outbreak were found to have ≤23 SNVs different from each other and a minimum of 84 SNVs different from isolates not associated with the outbreak, which included sporadic isolates, a concurrent smaller outbreak associated with a summer fair and a 2012 beef outbreak. The intra-outbreak SNV differences in the two other outbreaks were 0–5 SNVs and it should be noted that 109 of the 117 sequenced pork outbreak isolates were also 0–5 SNVs different from each other. The same isolates were also subjected to *k*-mer analysis in which the genome is segmented into nucleotide sequences of a pre-determined length (in this case 25-mers) and the frequency of each *k*-mer in the entire genome of each isolate is compared to the frequency of *k*-mers in all other isolates to determine a *k*-mer phylogeny tree. This analysis was able to cluster each outbreak into separate nodes. Interestingly, the *k*-mer method was also able to distinguish isolates from within the pork-associated outbreak that lacked some virulence genes present in all other outbreak isolates. The current surveillance methods and SNV analysis could not distinguish the isolates missing virulence genes from other isolates of the same outbreak. The difference between the two WGS methods is likely because SNV analysis compares conserved genomic regions where virulence factors are rarely found, whereas *k*-mer analysis analyzes the entire genome.

## WGS analysis approaches

The ideal bacterial typing method should have the following characteristics: accuracy, inter- and intra-laboratory reproducibility, stability with multiple passaging of isolates, high discriminatory power, concordance with epidemiological data, speed, ease-of-use, cost effectiveness, and amenability to computerized analysis (Van Belkum et al., 2007). WGS fulfills many of these requirements while providing better accuracy and discriminatory power than PFGE, MLVA and/or epidemiological investigations combined. WGS is currently being used by Public Health England for routine pathogen surveillance (Ashton et al., 2015), and PulseNet Canada is currently setting up a similar infrastructure. Before WGS becomes the international standard, especially for networks such as PulseNet, many issues still need to be addressed.

First of all, a comparison of the different software platforms needs to be performed against a set of isolates with known epidemiological and typing data. Although the studies discussed herein demonstrate that different pipelines can cluster outbreak isolates together with similar intra-outbreak SNV differences, the performance of each pipeline should be tested using the same set of isolates. Also, the SNV and *k*-mer analysis methods are very computer intensive (*k*-mer more so than SNV) and are not amenable to use by labs without the appropriate computing infrastructure or expertise. If SNV and/or *k*-mer methods were used as a standard, a new isolate would need to be compared to a curated regional, national and/or international database of isolates to place it into phylogenetic clusters, similar to viral genotyping. Other options such as whole-genome or core genome multi-locus sequence typing (wg or cgMLST), which look at most or all genes in an organism's genome, can create a barcode by assigning numbers for allelic variants and can be run on a desktop computer (Kohl et al., 2014; Leopold et al., 2014; Ruppitsch et al., 2015). Once a wgMLST database for *E. coli* is developed in PulseNet, it could become a strong contender for the standard WGS typing of STEC.

Once standards and issues surrounding the computing power and expertise are resolved, WGS will supplant current typing methods for STEC and most other organisms. It can also replace serotyping of STEC and typing of *stx* genes (Joensen et al., 2014, 2015) while providing the additional benefits of using the sequence for detecting virulence genes other than *stx* and providing data for research into genetic elements that influence pathogenicity. Finally, if the methods to perform metagenomics on stool are refined, WGS may also replace PCR or selective agars as the initial screening mechanism for STEC and other enteric pathogens. However, there remain significant technical, economic and organizational hurdles to overcome, before the practical use of genome-wide typing and analysis approaches in routine STEC investigations become a reality (Köser et al., 2012; Franz et al., 2014).

## Experiences at the alberta ProvLab

There have been continuous improvements in STEC detection through bacterial culturing, immunochemistry, and molecular and genomic methods. The advancements in each of these methods aim to increase assay sensitivity, specificity, speed, throughput and broad-ranging strain inclusivity. Despite the improvements in commercial assays and technologies, the adoption of detection methods that encompass non-O157 STEC serotypes by frontline laboratories has been slow. For this reason, there is likely to be a continued significant underreporting of STEC infections in current surveillance data in many countries. At the Alberta ProvLab, multiple studies of regional STEC rates and serotypes revealed that a diverse range of serotypes exist among non-O157 STEC in the province; 99% of these were identified by culture method and included O157 (*n* = 99), top six (*n* = 45), and non-top six (*n* = 36) (Chui et al., 2011, 2015a,b; Couturier et al., 2011). Most of the non-O157 STEC identified would not have been detected by routine frontline testing, which is restricted to detect only O157 STEC. Notably, between 8.3 and 52.9% of the non-O157 serotypes identified in these studies were positive for Stx2. Therefore, these findings reveal the underreporting of non-O157 STEC and the potentially pathogenic strains that risk being undetected in the population (Couturier et al., 2011; Chui et al., 2011, 2015a,b; Gould et al., 2013).

## Conclusion

There has been much dialog surrounding strategies for more comprehensive STEC identification by frontline laboratories. Primarily these approaches focus on the inclusion of Stx typing in routine testing. More recently, the development and incorporation of WGS methods in STEC surveillance aim to improve the epidemiological tracking of infections. As a secondary benefit of adopting WGS, will be a enhancement in our understanding of STEC biology through the vast collection of genome sequences. Although, there is great promise in WGS in STEC characterization and surveillance, STEC detection will likely continue to rely on a combination of culturing and non-culture methods. As such, regardless of the technologies that arise for STEC detection and characterization, for at least the immediate future, frontline laboratories will continue to need logical testing algorithms that incorporate a selection of the appropriate methods above.

## Author contributions

All authors listed, have made substantial, direct and intellectual contribution to the work, and approved it for publication.

## Funding

Salary support for BP was provided through Alberta Innovates - Health Solutions (AIHS) funding of The Alberta Provincial Pediatric EnTeric Infection Team.

### Conflict of interest statement

The authors declare that the research was conducted in the absence of any commercial or financial relationships that could be construed as a potential conflict of interest.

## Footnotes

^1^Thermo Fisher Scientific, Waltham, USA IonTorrentTM, Thermo Fisher Scientific. Available online at: https://www.thermofisher.com/ca/en/home/brands/ion-torrent.html (Accessed January 12, 2016).

^2^Illumina Inc., San Diego, USA Illumina Sequencing Platform Systems Portfolio. Available online at: http://www.illumina.com/content/dam/illumina-marketing/npiassets/PDF/brochure-sequencing-systems-portfolio.pdf (Accessed January 12, 2016).

^3^Applied Maths NV, Sint-Martens-Latem, Belgium BioNumerics Seven: a unique software platform | Applied Maths. Available online at: http://www.applied-maths.com/bionumerics (Accessed January 12, 2016).

^4^Ridom GmbH, Münster, Germany Ridom SeqSphere+. Available online at: http://www.ridom.de/seqsphere/u/User_Guide.html (Accessed January 12, 2016).
